# Supplementary material for: Cisplatin-Loaded M1 Macrophage-Derived Vesicles Have Anti-Cancer Activity in Osteosarcoma
Source: Cells. 2025 Oct 17;14(20):1616. doi: 10.3390/cells14201616 (PMC12564448; doi:10.3390/cells14201616)
Supplement: Supplementary file 1 [file cells-14-01616-s001.zip › cells-3892018-supplementary.pdf]

Cisplatin-Loaded M1 Macrophage-Derived Vesicles Have Anti-Cancer Activity in Osteosarcoma

Namrata Anand [1,2], Joseph Robert McCorkle [1], David S. Schweer [1,3], Lan Li [4], Kristen S. Hill [1], Melissa A. Fath [5], Derek B. Allison [6], Christopher L. Richards [4] and Jill M. Kolesar [5,*]

[1] Markey Cancer Center, College of Medicine, University of Kentucky, Lexington, KY 40536, USA; namrata.anand@bsd.uchicago.edu (N.A.); rob.mccorkle@uky.edu (J.R.M.); schweerds@arizona.edu (D.S.S.); kristen.hill@uky.edu (K.S.H.)
[2] Department of Pharmacy Practice and Research, College of Pharmacy, University of Kentucky, Lexington, KY 40508, USA
[3] Department of Obstetrics & Gynecology, College of Medicine, University of Kentucky, Lexington, KY 40508, USA
[4] Department of Chemistry, College of Arts and Science, University of Kentucky, Lexington, KY 40506, USA; lan.li@uky.edu (L.L.); chris.richards@uky.edu (C.L.R.)
[5] Department of Pharmaceutical Sciences and Experimental Therapeutics, College of Pharmacy, University of Iowa, Iowa City, IA 52242, USA; melissa-fath@uiowa.edu
[6] Department of Pathology & Laboratory Medicine, College of Medicine, University of Kentucky, Lexington, KY 40506, USA; derek.allison@uky.edu

* Correspondence: jill-kolesar@uiowa.edu

Supplementary Figure files.

Supplemental Figure 1.

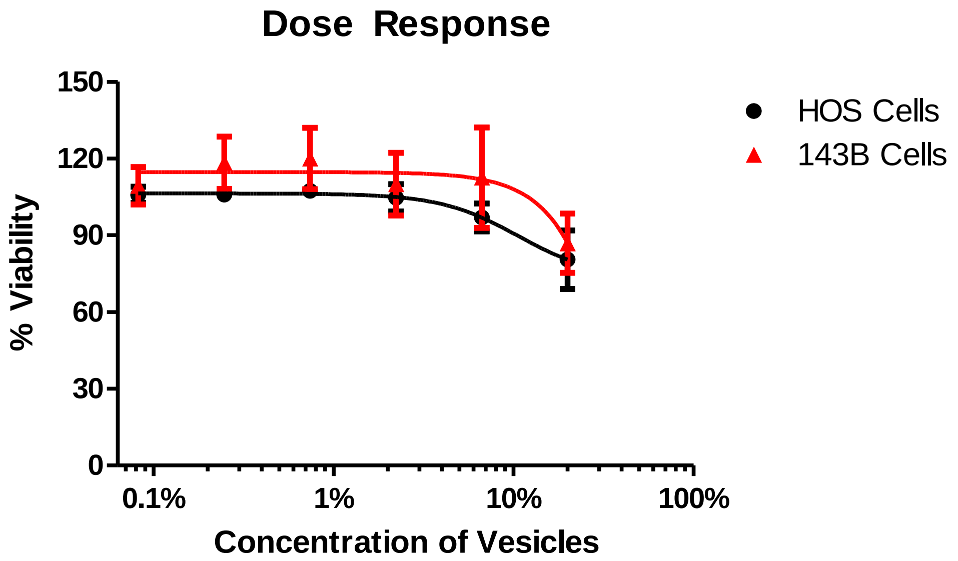

Figure S1. Dose-response curve of hE-MVs on HOS and 143B cells. HOS and 143B showed limited sensitivity towards hE-MVs. The dose-response curve was generated from three independent replicates of experiments performed in duplicates. The red plotted line indicates 143B cells and the black line represents HOS cells. (n=3, ± SEM, p=0.03 in HOS cells, *p=0.06* in 143B, Student's unpaired one-tailed t-test)

Supplemental Figure 2

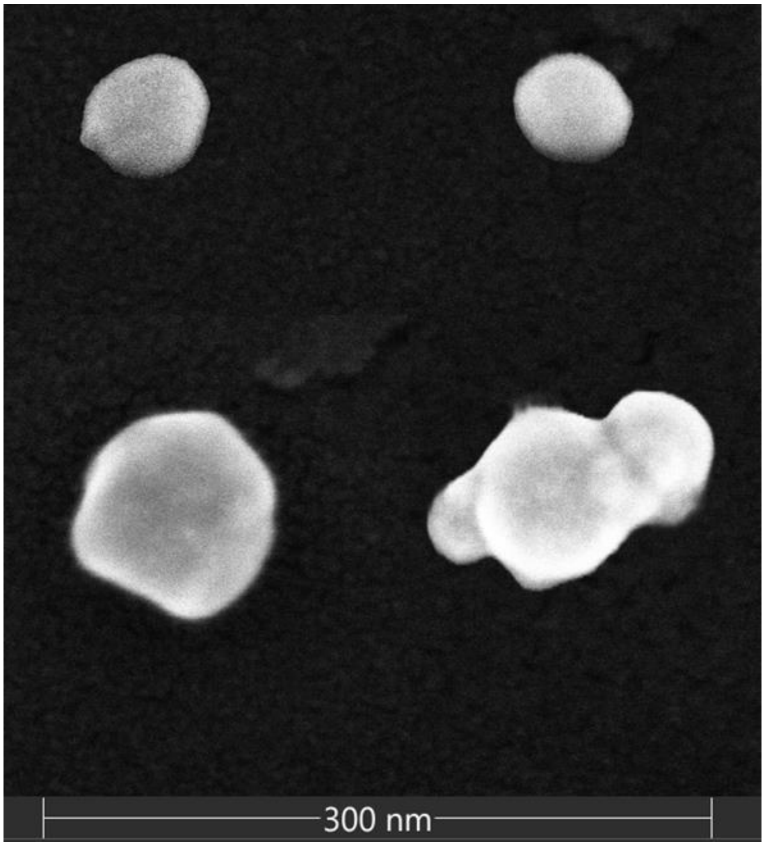

Figure S2. Field emission SEM of mE-MVs cells made using nitrogen cavitation.

Supplemental Figure 3

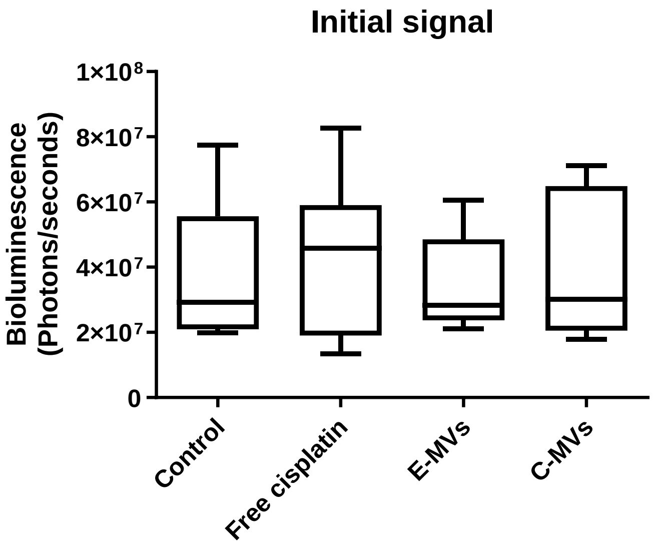

Figure S3. Initial bioluminescence radiance of mice across different groups of mice. Mice were randomized after reaching the desired bioluminescence and were divided into four different treatment groups. No significant difference was found between the groups.

Supplemental Figure 4.

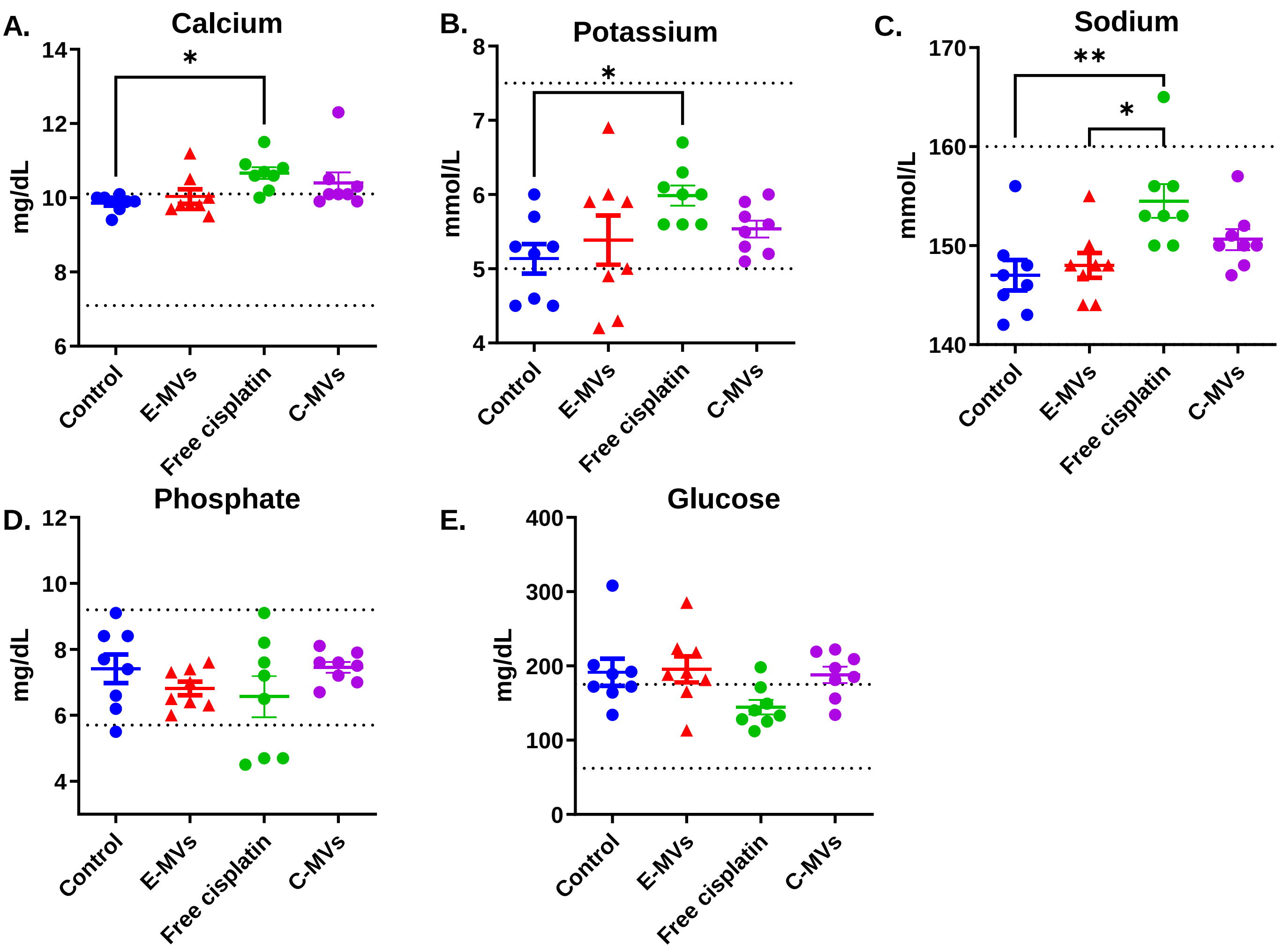

Figure S4. Assessment of serum electrolytes. A. Calcium levels were significantly higher in the free cisplatin group compared to the control. B. Potassium levels were significantly higher in the free cisplatin group vs control mice. C. Sodium levels were significantly higher in free cisplatin mice compared to control and mE-MVs mice. D&E. Phosphate and glucose levels were not significantly different between groups. p values were calculated using One–way ANOVA *$p<0.05$*, **$p<0.01$*. Data were presented as mean ± SEM, n=32, 8 groups/mice.

Supplemental Figure 5.

A.

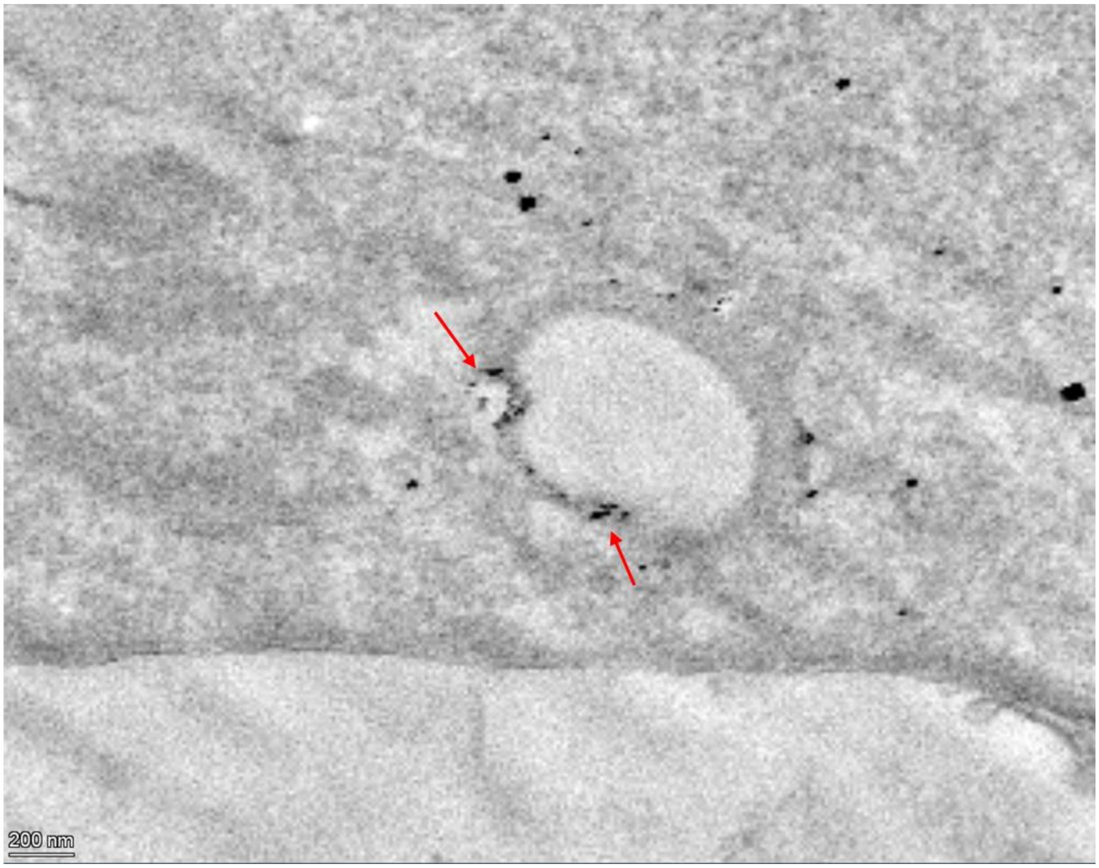

B.

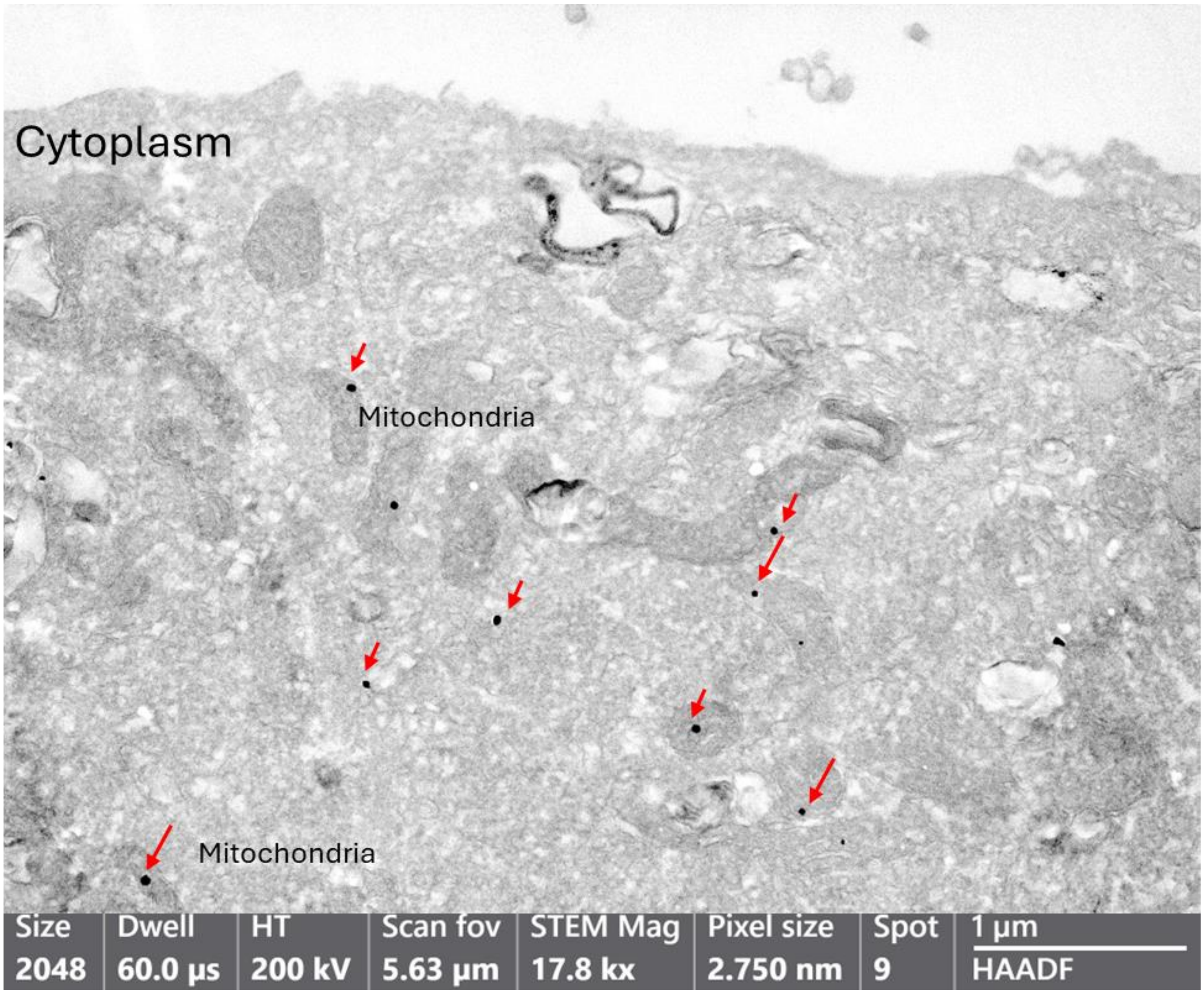

Supplemental Figure 5. Transmission electron microscopy of hMVs internalized in a 143B cell. hMVs labeled with gold nanoparticles (tiny black dots shown with red arrows).
